# A Randomized Controlled Study Comparing Reverse Hybrid Therapy and Standard Triple Therapy for *Helicobacter pylori* Infection

**DOI:** 10.1097/MD.0000000000002104

**Published:** 2015-12-07

**Authors:** Ping-I Hsu, Sung-Shuo Kao, Deng-Chyang Wu, Wen-Chi Chen, Nan-Jing Peng, Hsien-Chung Yu, Huay-Min Wang, Kwok-Hung Lai, Jin-Shiung Cheng, Angela Chen, Seng-Kee Chuah, Feng-Woei Tsay

**Affiliations:** From the Division of Gastroenterology, Kaohsiung, Taiwan (P-IH, S-SK, W-CC, H-CY, H-MW, K-HL, J-S C, F-WT); Department of Internal Medicine, Kaohsiung, Taiwan; Department of Nuclear Medicine, Kaohsiung, Taiwan (N-JP); Kaohsiung Veterans General Hospital and National Yang-Ming University, Kaohsiung, Taiwan; Division of Gastroenterology, Kaohsiung, Taiwan (D-CW); Department of Internal Medicine and Cancer Center, Kaohsiung Medical University Hospital, Kaohsiung, Taiwan; Department of Medicine, Kaohsiung, Taiwan (D-CW); Faculty of Medicine, College of Medicine, Kaohsiung Medical University, Kaohsiung, Taiwan; Institute of Biomedical Sciences, Kaohsiung, Taiwan (AC), National Sun Yat-Sen University, Kaohsiung, Taiwan; Division of Hepato-Gastroenterology, Kaohsiung, Taiwan (S-KC); and Department of Internal Medicine, Kaohsiung Chang Gung Memorial Hospital, Kaohsiung, Taiwan; Chang Gung University College of Medicine, Kaohsiung, Taiwan.

## Abstract

Reverse hybrid therapy is an 1-step 2-phase treatment for *Helicobacter pylori* (*H. pylori*) infection with less cost than standard triple therapy. We conducted a randomized, controlled study to compare the efficacies of standard triple therapy and reverse hybrid therapy in the treatment of *H. pylori* infection.

From October 2012 to March 2015, consecutive *H. pylori*-infected subjects were randomly allocated to receive either a reverse hybrid therapy (pantoprazole plus amoxicillin for 12 days and clarithromycin plus metronidazole for the initial 7 days) or a standard triple therapy (pantoprazole plus amoxicillin and clarithromycin for 12 days). *H. pylori* status was assessed 6 weeks after treatment. Additionally, antibiotic resistances and host *CYP2C19* genotypes were examined and analyzed.

A total of 440 *H. pylori*-infected patients were randomly assigned to receive either a reverse hybrid (n = 220) or a standard triple therapy (n = 220). The reverse hybrid group had a higher eradication rate than standard triple group either by intention-to-treat (93.6% vs. 86.8%; *P* = 0.016) or per-protocol analysis (95.7% vs. 88.3%; *P* = 0.005). The 2 patient groups exhibited similar frequencies of overall adverse events (14.1% vs. 9.5%) and drug compliance (96.8% vs. 98.6%). Clarithromycin resistance was an independent risk factor predicting eradication failure in standard triple group (*P* < 0.001), but not in reverse hybrid group. *CYP2C19* genotypes did not affect the eradication rates in both groups.

Reverse hybrid therapy can be considered for first-line treatment of *H. pylori* infection since the new therapy achieves a higher eradication rate than standard triple therapy with similar tolerability and less pharmaceutical cost.

## INTRODUCTION

*Helicobacter pylori* (*H. pylori*) infection is well recognized as the leading cause of chronic gastritis, peptic ulcer disease, and gastric cancer.^1–5^ Currently, *H. pylori* treatment remains a challenge for physicians as antimicrobial resistance has continued to increase worldwide. Although standard triple therapy has been recommended as the first-line therapy in many guidelines,^6–9^ its eradication rate has decreased to unacceptable level in most parts of the world.^10–15^ The growing treatment failure rate is generally attributed to an increasing prevalence of resistance to clarithromycin, a basic component of standard triple therapy.^10–12,15^ An updated consensus report^8^ has therefore proposed a bismuth-containing quadruple therapy or non-bismuth quadruple therapy (sequential or concomitant therapy)^16–18^ as first-line treatment in settings with clarithromycin resistance rates greater than 15% to 20%. Although levofloxacin-based triple therapy can achieved a high eradication rate in populations with clarithromycin resistance greater than 20% and quinolone resistance less than 10%,^19^ it is not generally recommended as a first-line therapy on concerns of the rapid development of resistant strains.

Hybrid (dual–quadruple) therapy developed by Hsu et al. consists of a proton pump inhibitor (PPI) and amoxicillin for 14 days with addition of clarithromycin and metronidazole for the final 7 days.^20^ In its pilot study, hybrid therapy generated excellent eradication rates of 99% and 97% according to per-protocol (PP) and intention-to-treat (ITT) analyses. Subsequent randomized trials demonstrated that hybrid regimens were either comparable with or more effective than sequential therapies.^21–23^ Recently, a large multicenter randomized trial also showed that both 14-day hybrid and 14-day concomitant therapies cured more than 90% of the patients with *H. pylori* infection in areas with high clarithromycin and metronidazole resistance.^24^ Many experts have recommended hybrid therapy as a treatment option for *H. pylori* in areas with moderate or high clarithromycin resistance.^25–27^

Recently, we further tested whether the duration of hybrid therapy could be reduced while maintaining the high eradication rate.^28^ Two hundred twenty *H. pylori*-infected subjects were randomized to receive a 10-, 12-, or 14-day hybrid therapy. The eradication rates with PP analyses were similar: 95.0% for 10-day, 95.1% for 12-day, and 93.4% for 14-day hybrid therapies.^28^ The results suggested that the duration of hybrid therapy could be reduced to 12 days or less while maintaining the high eradication rate.

**FIGURE 1:**
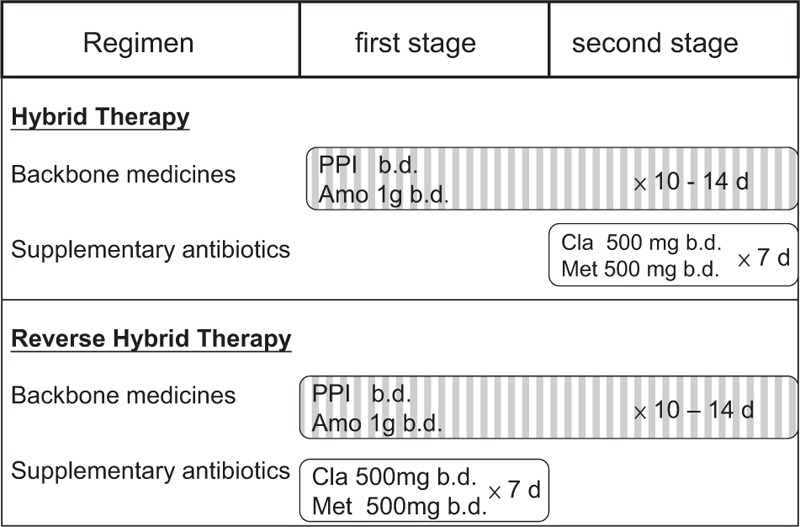
Hybrid regimen and reverse hybrid regimen. Hybrid therapy (a 2-step 2-phase therapy) consists of a proton pump inhibitor and amoxicillin (backbone drugs) for 10 to 14 days, with addition of clarithromycin and metronidazole for the final 7 days; reverse hybrid therapy (an 1-step 2-phase therapy) consists of a proton pump inhibitor and amoxicillin for 10 to 14 days, with addition of clarithromycin and metronidazole for the first 7 days.

From the perspective of clinical practice, reversing the drug administration sequence in hybrid therapy (a quadruple regimen followed by a dual regimen) simplifies the treatment and makes it an 1-step 2-phase therapy (Fig. 1). Although whether changing the administration sequence would reduce the efficacy of hybrid therapy is unclear, 1 of our recent studies demonstrated similar efficacies of 10-day reverse and standard sequential therapies in the treatment of *H. pylori* infection.^29^

Currently, whether hybrid therapy is superior to standard triple therapy remains unanswered. To address this issue, we conducted this multicentre, randomized controlled trial to compare the efficacies of 12-day reverse hybrid therapy and 12-day standard triple therapy. Furthermore, we also investigated the impact of antibiotic resistance and polymorphism of *CYP2C19* on the eradication rates of the 2 first-line treatments.

## METHODS

### Participants

This study was a multicentre, single-blind, randomized trial conducted in conducted in the gastroenterology clinics at the Kaohsiung Veterans General Hospital, Kaohsiung Medical University Hospital, and Kaohsiung Municipal Hsiao-Kang Hospital in Taiwan between October 2012 and March 2015. The study was approved by the Institutional Review Board of each hospital. It was registered at ClinicalTrials.gov, trial NCT02359435.

All consecutive *H. pylori*-infected adult patients with endoscopically proven peptic ulcer diseases or gastritis were eligible for recruitment. The diagnosis of *H. pylori* was based on at least 2 positive results of rapid urease test, histology, and culture. Exclusion criteria were as follows: age younger than 20 years; prior *H. pylori* eradication, allergy to any of the medications used in the trial, presence of severe comorbidities (for example, decompensated liver cirrhosis, uremia), ingestion of antibiotics or bismuth within the prior 4 weeks, and pregnancy.

### Randomization

Using a computer-generated number sequence, we randomly allocated patients at a 1:1 ratio to either a 12-day reverse hybrid therapy (pantoprazole 40 mg plus amoxicillin 1 g twice daily for 12 days, and clarithromycin 500 mg plus metronidazole 500 mg twice daily for the first 7 days) or a 12-day standard triple therapy (pantoprazole 40 mg plus clarithromycin 500 mg and amoxicillin 1 g twice daily for 12 days). All drugs were taken 1 hour before breakfast and dinner. An independent research assistant at the Kaohsiung Veterans General Hospital generated the computerized random number sequence. She prepared the study medicines and instructions for drug administration according to the number sequence and concealed them in an opaque envelope. The opaque envelopes labeled with sequence numbers outside were kept by research nurses at each study hospital.

After written informed consent was obtained from each participant, the independent research assistant at the Kaohsiung Veterans General Hospital reported by telephone each patient's treatment allocation to the research nurses at each study hospital. The patients took medicines according to the instructions in the envelopes. Both physicians and research nurses were blind to the treatment arm.

Patients were followed in outpatient clinics to investigate adverse effects and drug compliance at the second week. An additional 4-week pantoprazole (40 mg once daily) therapy was administered following eradication treatment for the patients with peptic ulcer disease. In contrast, the patients with gastritis only received 4-week antacid therapy following *H. pylori* eradication.

### Trial Design

Participants completed a standard questionnaire which contained questions regarding demographic data and personal history of smoking and alcohol consumption. For the analysis of *CYP2C19* genotypes, blood sampling was performed on enrollment. DNA was extracted from the leucocytes with a commercially available kit (Qiagen K.K., Tokyo, Japan). Genotyping procedures for identifying the *CYP2C19* wild-type gene and 2 mutated alleles, *CYP2C19* m1 and *CYP2C19* m2, were performed by polymerase chain reaction-based restriction fragment length polymorphism as previous description.^30^ Genotypes were classified into 3 distinct groups: homogeneous extensive metabolizer (homEM); heterogeneous extensive metabolizer (hetEM); poor metabolizer (PM).

Adverse events were assessed by a specific questionnaire fulfilled at the end of eradication therapy. The severity of adverse events were recorded according to a 4-point (none; mild; moderate; severe) scale system as previous description.^31^ Compliance with therapy was determined via pill counts.

Patients with gastric ulcer on enrollment received a follow-up endoscopy with rapid urease test, histological examination and culture 6 weeks after the end of eradication therapy. In contrast, patients with gastritis or duodenal ulcer underwent a urea breath test to assess final *H. pylori* status. Cure of *H. pylori* infection was defined as a negative result of urea breath test, or negative results of all histology, urease test, and bacterial culture.^32^

Because of facility problems, *H. pylori* culture was only conducted in the Kaohsiung Veterans General Hospital. We took a specimen from the antrum for culture of *H. pylori*.^33^ and then rubbed the specimen on the surface of an agar plate (Campy-BAP agar plate; Brucella agar + 10% whole sheep blood + IsoVitalex). The agar plate was incubated at 37°C with microaerobic condition for 5 days. If one or more colonies of Gram-negative bacilli were positive in urease, catalase, and oxidase tests, the culture result was considered positive. The E-test (AB Biodisk, Solna, Sweden) was used to evaluate the resistance to antibiotics according to minimum inhibitory concentration (MIC) values of >1, >0.5, and >8 μg/mL for clarithromycin, amoxicillin, and metronidazole, respectively.^34^

### Statistical Methods

Eradication rate was the primary outcome of this study. It was evaluated by ITT, modified ITT and PP analyses. ITT analysis included all eligible patients enrolled in the study regardless of compliance with the study protocol. Patients with unavailable data following treatment were assumed to have been treated unsuccessfully. Modified ITT analysis included all those receiving at least 1 dose of drug and undergoing follow-up tests for *H. pylori* infection. PP analysis only included patients with good drug compliance and excluded patients with unknown *H. pylori* status following therapy. The second outcomes were the frequency of adverse events and drug compliance. Compliance was defined as good (taking at least 80% of eradication medication) or poor (taking less than 80% of the total medication). Differences in demographic data, eradication rates, and adverse events among different arms were determined by Chi-square test or Fisher exact test, as appropriate. The Student *t* test was used for the comparison of continuous data. SPSS (version 12.0 for Microsoft Windows) were used for all statistical analyses. A *P*-value of <0.05 was considered statistically significant.

According to previous studies,^35^ the eradication rate of 12-day standard triple therapy was 85%. It was estimated that at least 220 participants in each group were required to achieve a statistic power of 90% with a type I error of 0.05, assuming a 15% loss to follow-up and 4% of participant with poor medication compliance.

To identify the independent factors determining the eradication rate, clinical, genetic, and bacterial parameters were initially analyzed by univariate analysis. The parameters with significant differences in univariate analysis were then further analyzed by a logistic regression method to search the factors independently predicting eradication rate.

## RESULTS

### Patients

**FIGURE 2:**
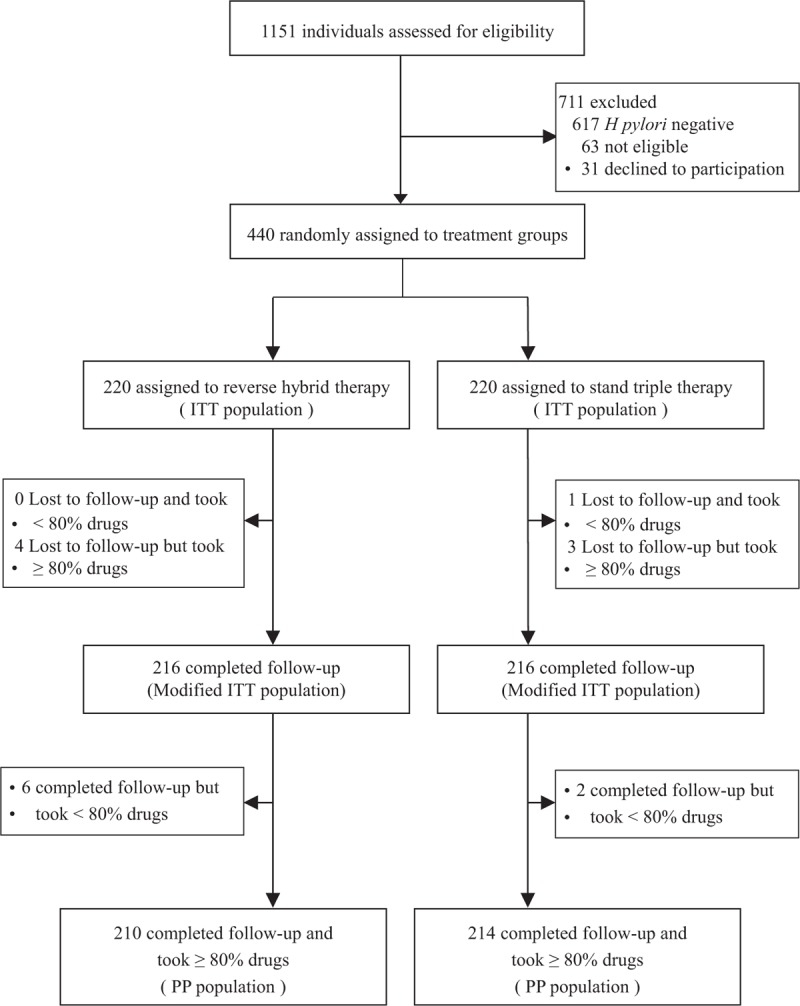
Trial profile. ITT = intention-to-treat; PP = per-protocol.

From October 2012 to March 2015, a total of 440 *H. pylori*-infected patients were recruited for the study and randomly allocated to the reverse hybrid (n = 220) or standard triple group (n = 220). The baseline demographic and clinical characteristics of patients in both groups are shown in Table 1. There were no differences in all parameters between groups. The flow of patients through the study is shown in Figure 2. In the reverse hybrid therapy group, 10 participants were excluded from PP analysis for loss to follow-up (n = 4) or poor compliance (n = 6). In the standard triple therapy group, 6 participants were excluded from PP analysis for loss to follow-up (n = 3), poor compliance (n = 2), or both (n = 1).

**TABLE 1:**
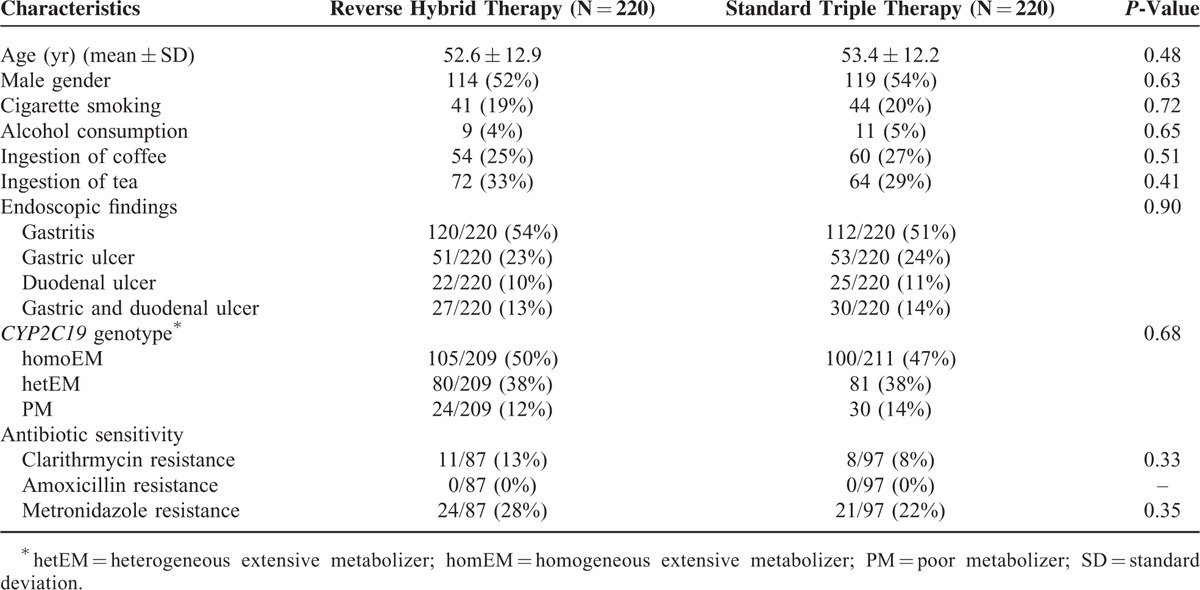
Participant Characteristics by Treatment Group

### Eradication of *H. pylori*

The outcomes of treatments are listed in Table 2. The ITT eradication rates were 93.6% (95% confidence interval [CI], 90.4–96.8%) and 86.8% (95% CI, 82.3–91.3%) for reverse hybrid and standard triple therapies, respectively. Reverse hybrid therapy achieved a higher eradication rate than standard triple therapy (95% CI, 1.3–12.3%; *P* = 0.016). The modified ITT (95.4% vs. 88.4%) and PP analyses (95.7% vs. 88.3%) yielded similar results (*P* = 0.008 and 0.005, respectively).

**TABLE 2:**
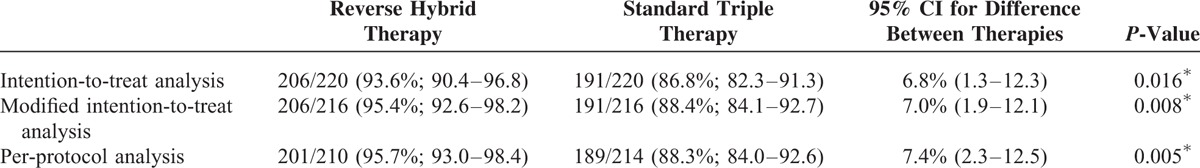
Eradication Rates in the ITT and PP Populations

### Eradication Rates in Resistant Strains

*H. pylori* culture was performed in 230 patients at initial endoscopy. *H. pylori* strains were isolated from 184 (80.6%) of them. Antibiotic resistance rates in each group are listed in Table 1. The overall clarithromycin, amoxicillin and metronidazole resistant rates of *H. pylori* strains isolated in this trial were 10.3% (19/184), 0.0% (0/184) and 24.5% (45/184), respectively. Reverse hybrid therapy achieved a higher eradication rate for clarithromycin-resistant strains than standard triple therapy (90% vs. 25%, *P* = 0.01). However, the 2 therapies had comparable eradication efficacy for clarithromycin-sensitive strains (99% vs. 97%). There were no differences in treatment efficacy between the 2 therapies for either metronidazole-sensitive (100% vs. 93%) or resistant strains (91% vs. 81%). In the reverse hybrid therapy group, the eradication rates of the strains with nonresistance, single clarithromycin resistance, single metronidazole resistance, and dual resistances were 100%, 100%, 95%, and 67%, respectively. The corresponding eradication rates in the standard therapy were 99%, 33%, 90%, and 0%, respectively. Reverse hybrid therapy had a higher eradication rate for single clarithromycin-resistant strains than standard triple therapy (*P* = 0.021).

### Adverse Events and Compliances

The incidences of adverse events in the participants receiving reverse hybrid and standard triple therapies were 14.1% (95% CI, 9.2–19.0%) and 9.5% (95% CI, 5.6–13.4%), respectively. The 2 therapies exhibited similar frequencies of overall adverse events (*P* = 0.14).

Table 3 lists the profiles of adverse events of the 2 eradication therapies. Nausea was the most common adverse event in all the 2 treatment groups (7.3% and 2.7% in reverse hybrid and standard triple groups, respectively). The former had a higher frequency of nausea than the latter (*P* = 0.03). There were no significant differences in the frequencies of other adverse events between groups. Three patients in the hybrid group stopped the anti-*H. pylori* medication because of abdominal pain (n = 1), headache (n = 1), and dizziness (n = 1). In the standard triple group, 3 patients discontinued treatment owing to diarrhea (n = 1), dizziness (n = 1), and skin rash (n = 1). Reverse hybrid and standard triple groups displayed similar compliance rates (96.8% [95% CI, 94.5–99.1%] and 98.6% [95% CI, 97.1–100.2%], respectively).

**TABLE 3:**
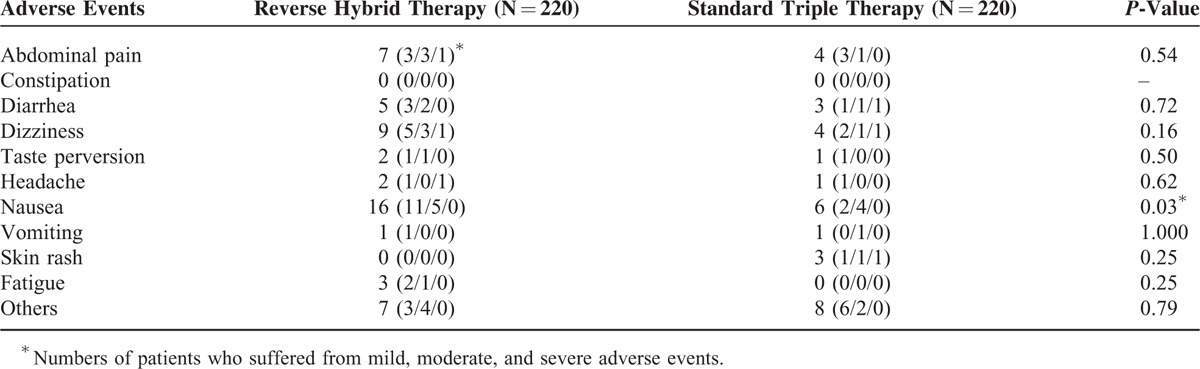
Adverse Events of Reverse Hybrid and Standard Triple Therapies

### Factors Influencing Efficacy of Anti-*H. pylori* Therapy

Univariate analysis for the clinical, host, and bacterial factors did not identify any risk factors associated with treatment failure by reverse hybrid therapy (Table 4). The eradication rate of standard triple therapy was affected by the history of smoking (*P* = 0.033), presence of peptic ulcer (*P* = 0.002), and clarithromycin resistance (*P* < 0.001), but not by host *CYP2C19* genotype and other clinical factors (Table 4). Multiple regression analysis showed that clarithromycin resistance was the only independent factor predicting eradication failure with an odds ratio of 209.6 (95% CI, 10.8–4082.3; *P* < 0.001).

**TABLE 4:**
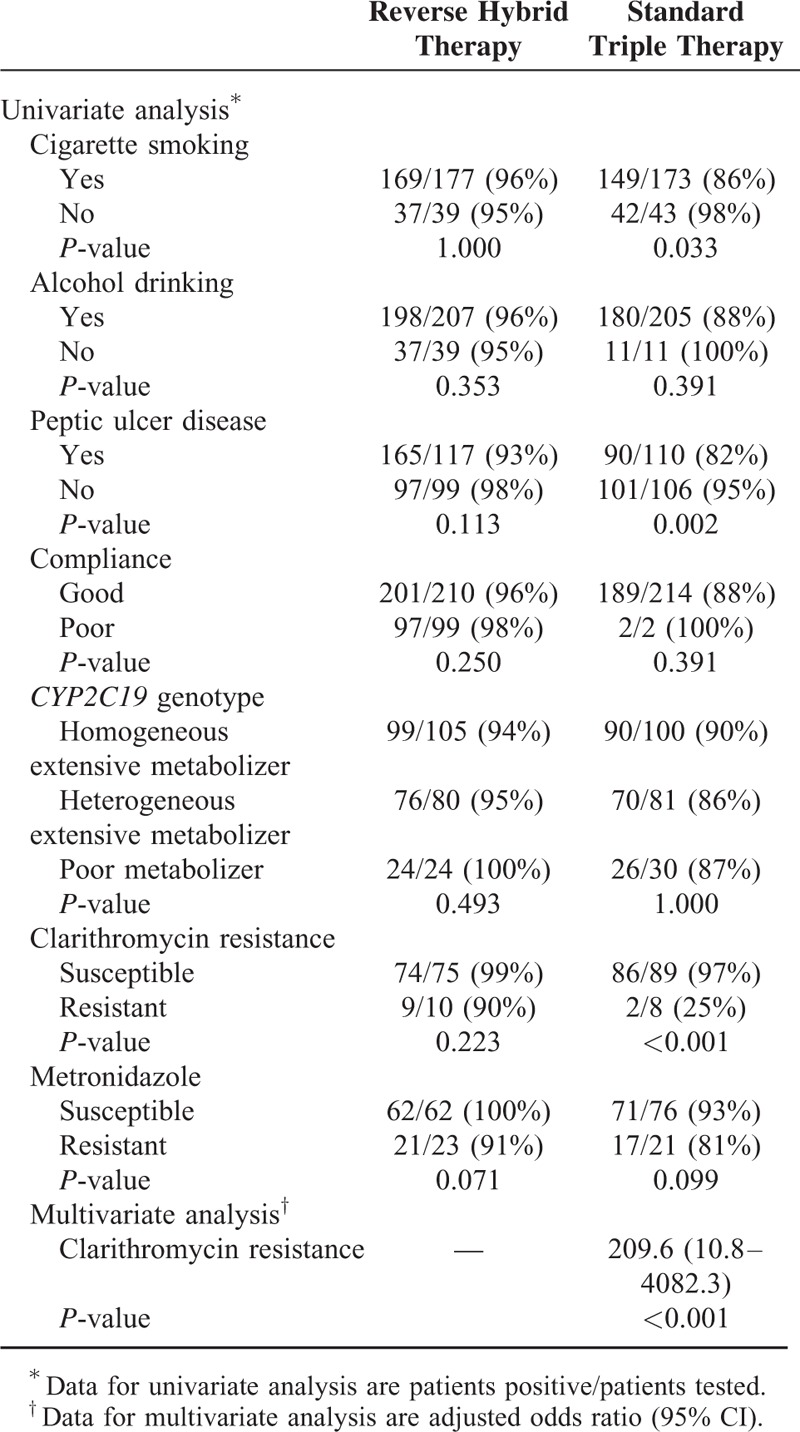
Factors Affecting Eradication in Reverse Hybrid and Standard Triple Therapies

## DISCUSSION

With the rising prevalence of antimicrobial resistance, the treatment success of standard triple therapy has recently declined to less than 80% in many countries. An ideal treatment for *H. pylori* infection should be highly effective, well tolerated, simple, and inexpensive. This study is the first to conduct a head-to-head, randomized, controlled trial to assess the efficacy of 12-day standard triple therapy and that of 12-day reverse hybrid therapy for *H. pylori* eradication. The results clearly showed that 12-day reverse hybrid therapy achieved a higher eradication rate than 12-day triple therapy, whether by ITT (93.6% vs. 86.8%), modified ITT (95.4% vs. 88.4%), or PP analyses (95.7% vs. 88.3%). The 2 treatments were well tolerated and shared comparable total adverse events (14.1% and 9.5%) and compliance (96.8% and 98.6%). With regard to pharmaceutical cost, 12-day reverse hybrid therapy was cheaper than 12-day triple therapy (0.37.2 vs. 0.44.0 in Taiwan). The data support the use of reverse hybrid therapy as the standard first-line treatment for *H. pylori* infection.

As a general rule for the treatment of other infectious diseases, clinicians should prescribe therapeutic regimens that have a PP eradication rate ≥90% for anti-*H. pylori* therapy.^10,25–27,35^ Numerous previous reports^11–13^ disclosed that clarithromycin resistance is the key factor determining the treatment efficacy of standard tripe therapy. According to the theoretical effect of increasing clarithromycin resistance on the success of standard triple therapy, PP eradication rate falls to 90% with 5% clarithromycin resistance for 7-day therapy and 15% resistance for 14-day therapy.^36^ Two recent randomized controlled trials confirmed that the 7-day standard triple therapy had a cure rate of 82% in an area with 18% clarithromycin resistance,^18^ and 14-day standard triple therapy achieved an eradication rate of 87% in an area with 11% clarthromycin resistance.^37^ In the present study, the clarithromycin resistant rate of *H. pylori* strains in the study population was 10.3%. The 12-day standard triple therapy failed to surpass a 90% eradication rate (only 88 3% by PP analysis). In contrast, 12-day reverse hybrid therapy achieved an eradication rate ≥95% (95.7% by PP analysis). Molina-Infante and his colleagues^24^ also confirmed that 14-day hybrid therapy cured more than 90% of *H. pylori* infections in areas with high clarithromycin (24%) and metronidazole resistance (34%). The aforementioned data strongly indicate that reverse hybrid therapy can replace the standard triple therapy as the first-line treatment for *H. pylori* infection in areas with clarithromycin resistance ≥10% since the new therapy achieves a higher eradication rate than standard triple therapy with similar tolerability and less pharmaceutical cost.

Many antibiotics applied in *H. pylori* eradication therapy are acid-sensitive. PPIs can increase the activity of some antibiotics by reducing gastric acid secretion and possess direct anti-*H. pylori* activity.^38^ Most of them are metabolized by the hepatic cytochrome P450 system, especially CYP2C19.^38^ Several previous reports demonstrated that the *CYP2C19* homEM genotype was an independent factor determining the success rate of *H. pylori* eradication therapy.^38,39^ In this study, the subjects with *CYP2C19* homEM genotype had a lower eradication rate than those with PM genotype (100% vs. 94%) in the reverse hybrid therapy group. However, the difference did not reach statistical significance. In the stand triple therapy group, there were also no significant differences in eradication rates among the subjects with homEM, hetEM, and PM genotypes.

To simplify eradication regimen, the sequence of drug administration in hybrid therapy was reversed in this study (Fig. 1). Patients took 4 drugs in the initial phase and then took the remaining 2 antibiotics in the second treatment phase. This altered drug administration sequence rendered the hybrid therapy an 1-step 2-phase treatment. Whether reverse hybrid (a 4 + 2 regimen) and standard hybrid therapies (a 2 + 4 regimen) achieve a similar eradication rate remains unclear. However, the eradication rates of 12-day reverse hybrid therapy in this study and 12-day standard hybrid therapy in one of our previous studies^28^ were comparable (95.7% vs. 95.1% by PP analysis). The data suggest that both reverse and standard hybrid regimens can achieve an eradication rate exceeding 90% in the first-line treatment of *H. pylori* infection.

Our study had several limitations. First, the number of *H. pylori* strains with antibiotic susceptibility data in either reverse hybrid or standard triple therapy group was too small to make a robust conclusion for the impacts of antibiotic resistances on the eradication rates of each therapy. Second, the study was conducted in a single country. The results are therefore needed to be confirmed in other countries with different patterns of antibiotic resistances. However, this study is the first trial comparing reverse hybrid therapy and standard triple therapy in the treatment of *H. pylori* infection. Additionally, it is a randomized-controlled trial with large sample size (>400 participants).

In conclusion, reverse hybrid therapy can replace the standard triple therapy as the first-line treatment for *H. pylori* infection in areas with clarithromycin resistance ≥10% since the new one-step therapy achieves a higher eradication rate than standard triple therapy with similar tolerability and less pharmaceutical cost.
